# Making long-term memories in minutes: a spaced learning pattern from memory research in education

**DOI:** 10.3389/fnhum.2013.00589

**Published:** 2013-09-25

**Authors:** Paul Kelley, Terry Whatson

**Affiliations:** ^1^Science + Technology in LearningWhitley Bay, UK; ^2^Life, Health and Chemical Sciences, The Open UniversityMilton Keynes, UK

**Keywords:** long-term memory, long-term potentiation, encoding, spaced learning, spaced retrieval, education methods

## Abstract

Memory systems select from environmental stimuli those to encode permanently. Repeated stimuli separated by timed spaces without stimuli can initiate Long-Term Potentiation (LTP) and long-term memory (LTM) encoding. These processes occur in time scales of minutes, and have been demonstrated in many species. This study reports on using a specific timed pattern of three repeated stimuli separated by 10 min spaces drawn from both behavioral and laboratory studies of LTP and LTM encoding. A technique was developed based on this pattern to test whether encoding complex information into LTM in students was possible using the pattern within a very short time scale. In an educational context, stimuli were periods of highly compressed instruction, and spaces were created through 10 min distractor activities. Spaced Learning in this form was used as the only means of instruction for a national curriculum Biology course, and led to very rapid LTM encoding as measured by the high-stakes test for the course. Remarkably, learning at a greatly increased speed and in a pattern that included deliberate distraction produced significantly higher scores than random answers (*p* < 0.00001) and scores were not significantly different for experimental groups (one hour spaced learning) and control groups (four months teaching). Thus learning per hour of instruction, as measured by the test, was significantly higher for the spaced learning groups (*p* < 0.00001). In a third condition, spaced learning was used to replace the end of course review for one of two examinations. Results showed significantly higher outcomes for the course using spaced learning (*p* < 0.0005). The implications of these findings and further areas for research are briefly considered.

## Introduction

Memory systems select from the thousands of stimuli in the environment those to encode permanently. Scientists have tried to understand long-term memory (LTM) processes through a variety of approaches including using repeated, spaced stimuli (Ebbinghaus, 1913; Pavlov, 2010). Pavlov and his associates focused on memory encoding of demonstrably new associations (a link between the sound of a bell and food), and Ebbinghaus on memory retrieval, testing himself with word-like sequences of nonsense syllables. For almost a century after these first studies there remained two intractable issues: first, what was the physical basis of LTM encoding, and second, how could it be triggered most effectively? (Fields, 2005, 2011).

Recently a robust model of LTM formation has emerged through studies of late Long-Term Potentiation (LTP) and LTM in many different contexts and species (Morris, 2003). These studies show repeated stimuli spaced by periods without stimuli can lead to intracellular signaling mechanisms activating genes, initiating the production of proteins (Scharf et al., 2002; Hernandez and Abel, 2008). These proteins then can strengthen sensitized synapses, triggering LTP and LTP encoding (Frey and Morris, 1997; Barco et al., 2008; Moncada et al., 2011). The effectiveness of spaced repetition in creating long-term memories has been experimentally demonstrated in many species in time scales of minutes (Itoh et al., 1995; Scharf et al., 2002; Morris, 2003). The LTP/LTM processes differentiate LTM from short-term memory (STM) as the processes of synaptic tagging and capture do not occur in STM. So, while STM creates temporary memories more rapidly, these memories quickly fade in a day or two; in contrast, LTM can last a lifetime.

In behavioral studies using rapid repetition, the length of the spaces between stimuli proved a critical variable in LTM encoding. This was examined in an ingenious study of honeybees contrasting massed and spaced learning (Menzel et al., 2001). Using spaces between stimuli of 30 sec, 3 min, and 10 min, memory retention was tested after 30 min, one day and three days. Honeybees trained with 30 sec spaces showed the best learning after 30 min with over 80% retention, but this rapidly decreased, falling to 20% on the third day, a demonstration that only STM had been created. In contrast, honeybees trained with 10 min spaces between learning trials showed less than 80% retention after 30 min but subsequently consolidated these memories, reaching almost 100% on the third day, demonstrating long-term memories had been created.

Later research demonstrated two waves of transcription are required for LTM in honeybees: an early transcription wave (triggered during conditioning) and another starting several hours after learning (Lefer et al., 2012), as well as consolidation of LTM during sleep (Beyaert et al., 2012). These post-conditioning LTM processes also occur in humans as long-term memories are also consolidated during sleep, and this has been demonstrated in a number of ways, including studies showing task performance can be increased without further training (Walker and Stickgold, 2010). Thus LTM encoding processes trigger consolidation after learning whereas STM processes do not, showing a key gating process in memory systems for selecting stimuli in the environment to encode permanently.

LTM processes have been demonstrated in great detail in Drosophila where spaced training produces stabilized LTM (Tully et al., 1994) and de novo protein synthesis showing cAMP response element-binding protein (CREB) dependent gene transcription (Perazzona et al., 2004). Further research demonstrated a gating mechanism for LTM formation through dopaminergic neurons (Plaçais and Preat, 2013). Drosophila memory processes have recently been visualized *in vivo* using Kaede, a green florescent protein, confirming earlier *in vitro* studies showing de novo protein synthesis after spaced training was required for normal LTM formation, and transcriptional activities of key genes were elevated after spaced (but not massed) training (Chen et al., 2012). This has led to a further study demonstrating the evolutionary gain in gating LTM encoding, as creating LTP demands high energy input, in contrast with STM (Plaçais and Preat, 2013). Taken together, these findings robustly demonstrate the underlying mechanisms for LTP and LTM *in vivo*, and memory formation in specific neurons.

Studies in mammalian memory processes *in vitro* rat hippocampus cells clarified the importance of neural impulse activity in triggering LTP first identified in studies of nervous system development (Itoh et al., 1995). Using a pattern of three stimuli separated by 10 min spaces opened voltage-sensitive calcium channels in the cell membrane, activating signaling pathways to the nucleus. The pattern of impulse firing was echoed in the patterns of calcium flashes, and these were visualized using calcium-sensitive dye and scanning laser confocal microscopy to track pulses of calcium influx following stimuli (Fields, 2005). These and other related studies demonstrated there was no need for a specific signaling molecule from the membrane to the nucleus, and demonstrated the spaced pattern of action potential activity led to CREB phosphorylation and DNA synthesis of *zif* 268, a gene associated with memory (Dudek and Fields, 2002; Bukalo and Fields, 2008). Parallel studies in the biochemistry of memory over many years have clarified LTP processes further (Baudry et al., 2011).

Subsequently studies on living rats showed patterns of action potential activity have a critical role in long-term synaptic change, providing a direct link between *in vitro* studies of protein-dependent LTP and behavioral studies of LTM retention (Shires et al., 2012). These temporal patterns in LTM encoding have recently been linked to specific molecular LTP processes in a time scale of minutes (Naqib et al., 2012), and, in humans, more rapidly in *f*MRI studies of face recognition (Xue et al., 2011). Broadly speaking, LTP and LTM encoding processes occur in time scales of seconds, minutes and hours.

Encoding memories has been the subject of *f*MRI studies showing memory encoding and memory retrieval processes occur in different parts of the human brain. A seminal study of encoding complex scenes of unfamiliar information demonstrated posterior temporal-lobe structures associated with declarative memories focused in the parahippocampal cortex, whereas memory retrieval for successfully remembered information in the anterior temporal-lobe region focused in the subiculum (Gabrieli et al., 1997). Yet there remain significant similarities between these two memory processes.

In retrieving memories time patterns are also important though time scales in retrieval practice are usually in weeks, months or even years. Retrieval studies by Ebbinghaus (1913) demonstrated the value of spaced practice (many short sessions) over massed practice (a single long session) in LTM. Further studies confirmed this retrieval spacing effect, and led to attempts to implement the spacing paradigm in education. Although the spacing effect in retrieval has been demonstrated in many subjects and educational contexts to be effective, it has rarely been systematically implemented in education (Dempster, 1988; Seabrook et al., 2005). Despite recent careful analysis of the temporal patterns demonstrating effective recall of word pairs and other tasks (Cepeda et al., 2006; Pavlik and Anderson, 2008; Cepeda et al., 2009) and despite specific programmes based on the approach (Carpenter et al., 2009, 2012; Sobel et al., 2011), this remains the case. Yet the importance of time patterns in both LTP/LTM encoding (as demonstrated in neuroscience) and retrieval practice (as demonstrated in psychology) strongly suggest there are significant applications in education of evidence-based time patterns from both research traditions. A review by the Institute of Education Sciences of spaced retrieval approaches suggested this may be because studies included few examples showing acquisition of complex bodies of structured information, or a clear educational function in critical areas of education accountability such as high-stakes testing (Pashler et al., 2007).

Applying research in LTP/LTM encoding to learning complex bodies of structured information in education raises fundamental issues of the time scales and time patterns in current educational practice. One of the core functions of education is creating long-term memories through academic courses. These courses are frequently linked to measuring LTM through high-stakes testing, and these tests are of crucial importance to educational institutions, particularly in countries with national testing. Currently most standard teaching of courses can be characterized as massed instruction of 45 min or more (lessons) within courses studied over long time scales (months to years). In contrast, there has been compelling evidence demonstrating humans and other species create LTM in very short time scales, and LTP/LTM encoding has consistently shown repetitions spaced with short intervals of minutes duration are effective and even required in some species for LTP/LTM.

Studies of LTP/LTM encoding have time patterns that reflect the speed neurological processes. These range from action potential firing (on a scale of milliseconds) to the kinetics of intercellular process in LTP for synaptic tagging and capture (on the scale of minutes). There are other related processes, such as STM, that have longer time limits of up to a day or two. Although these time scales might seem far too short for LTM encoding of complex information as viewed from an educational perspective, they are common in learning in humans and other species, and essential in education.

The complexity of rapid human communication systems can be usefully considered in comparison with communication in other species. The study of language in humans and song in birds is a well-known example to demonstrate that humans are not the only species to be able to learn in early life complex communication systems. For example, both speech and birdsong acquisition require learning perception and production of complex information in time scales of seconds or less (Doupe and Kuhl, 2008). Understanding complex acoustic signals, producing highly structured vocalizations with format frequencies that change rapidly (20–100 ms), and producing ordered strings of sound with brief silent intervals are all achieved by young children and birds. The nightingale is capable of LTM encoding of 200 different song types and improving learning skills with practice, moving from being tutored in songs by other nightingales to learning new songs when alone. Nightingales learn large vocal pattern repertoires, song strings and chunk information when learning. They can readily memorize and produce long song sequences after 15 repetitions when young and only five as adults. Surprisingly the number of song types in a sequence can increase from 20 to 60 without requiring more repetitions. Like humans, nightingale songs are structured information from very short sound units creating long song sequences, with individual variations (Hultsch and Todt, 2008). Where humans excel is in communication through speech—using rapid, complex systems of sounds and silence to transfer information at high speed in social environments.

Memory processes *in vivo* for humans on the scale of individual neurons demonstrate how complex information in brief, rapid communications can be encoded in LTM. A study of patients with severe epilepsy showed specific memory formation in single neurons, and was able to demonstrate aspects of memory mechanisms in free recall. Three sessions of 16 five-seconds-long memorable video clips were shown to patients. Subsequently, internally generated reactivation of single neurons involved in memory acquisition of a clip occurred during free recall of that clip, demonstrating localization of some aspects of memory in specific neurons (Gelbard-Sagiv et al., 2008). Later studies of patients shown a wide range of images found different groups of neurons were encoding both abstract and basic properties of the images. This simultaneous representation may serve to bind separate aspects of visual objects into a coherent percept. More widely, this suggests memory processes can encode in a way that structures complex information (Steinmetz et al., 2011; Tse et al., 2013).

In order to apply in education neuroscience and behavioral research on time patterns for LTP/LTM encoding, it is necessary to identify, develop and test specific methods, based on a time pattern demonstrating LTP/LTM encoding. This study reports on such a method derived directly from the research demonstrating LTM mechanisms of DNA synthesis at an intracellular level can be triggered using three stimuli spaced by two 10 min periods without stimulation (Frey and Morris, 1997; Menzel et al., 2001; Bukalo and Fields, 2008). This method, termed “Spaced Learning” following Menzel et al. (2001), was created and refined by educators with the help of neuroscientists and social scientists, and explored in a variety of learning environments. This study reports on the development of Spaced Learning and a series of experiments using Spaced Learning carried out over a five-year period.

Not surprisingly given this LTM research is so recent, the model of standard teaching of courses over long periods through massed instruction has not been compared to methods based on LTP/LTM encoding. If time patterns in education are broadly optimal, the much shorter time patterns in Spaced Learning would lead to little learning and poor test results. On the other hand, if neuroscience and behavioral research into LTP/LTM encoding is both correct and can be effectively applied in an educational context, then using Spaced Learning to encode long-term memories of an educational course might demonstrate learning with significant learning and test results. The study was designed to give a preliminary answer to determine which hypothesis is supported.

## Materials and Methods

The development of Spaced Learning over the last seven years has been in two stages: a formative stage creating the method and preliminary trials, and a second stage focused formal experiments and high-stakes testing. In the first stage, the challenges for teaching professionals posed by translating LTP/LTM encoding research into a practical method were complex and time consuming. Teachers in the research team approached the task by addressing whether such a technique could be created, how it could be deployed effectively, whether learning did occur and, if so, would it do so in different contexts. Teachers’ central long-term concern was to determine whether Spaced Learning could demonstrate significant learning as measured by test results.

Supported by social scientists and neuroscientists during the process, these preliminary issues were resolved by secondary biology teachers synchronizing their educational practices to LTM/LTP encoding time patterns and time scales. Teachers with less knowledge of plasticity and the basic neuroscience of LTM/LTP encoding received support from the lead teacher trainer. This training focused on the physical basis of LTM, and the reasons the time pattern used was chosen. This led to exploring rapid communication in speech, text and media. Analogies and case studies proved useful in this process. The principle of LTM encoding was easily grasped as analogies with conventional wisdom were well known (for example, STM accounting for students “who knew it when they left the lesson but had forgotten it next lesson”). Very rapid communication as a strategy in Spaced Learning, though evident in language, reading and media acquisition in children, was less intuitive. The fact that the high-stakes test in Biology required students to answer 36 questions in 30 min, where questions often had 50 words or more, or the time scales of the Scholastic Aptitude Tests used for university admissions were more powerful based on a principle of learning matching testing.

The contrast with their normal teaching was considerable in a number of areas. Repeating the same content three times in the same session, albeit with minor changes, was highly unusual but manageable. The lead neuroscientist recommended the two 10 min spaces were distractor activities, ideally physical activities to minimize possible interference in the synaptic tagging and capture processes putatively occurring. This was completely new for all the teachers, who developed a wide range of options such as basketball practice, juggling, and modeling with clay-like materials (some options trialled proved impractical, such as aerobic exercise). Consistently given instruction at the speed of rapid communication was demanding, and teachers delivered Spaced Learning in pairs on occasion. The speed of instruction sessions required careful planning and resources constructed specifically for Spaced Learning. A recommendation from an advising neuroscientist was to restrict the time scale from the start of an instruction element to the end of the subsequent space to 30 min to limit the risk of interference in LTP/LTM processes. This meant instruction elements were always 20 min or less. Spaced Learning as used in study was therefore three intensive instruction elements of the same content with minor variations each lasting 20 min or less (stimuli), spaced by two distractor activities of 10 min (spaces without the stimuli).

Preliminary implementation was carried out using Spaced Learning to supplement standard teaching in a variety of contexts: before any teaching (overviews of new topics for example) or after all teaching (replacing course reviews). Reflection on implementation led to a host of refinements to the method and associated resources, and was followed by a range of more formal trials. The preliminary data indicated Spaced Learning led to learning; intense instruction elements were not a barrier to understanding, and testing days, weeks and months later after a Spaced Learning session indicated long-term memories had been encoded.

Teachers reacted differently to Spaced Learning though some responses were common to many teachers. Many found Spaced Learning fun, different, and felt it was a positive learning experience for both teachers and learners. Negative responses included rejecting the underlying neuroscience out of hand, a concern about greater workload, and a fear of school inspectors judging Spaced Learning sessions harshly because they did not match recommended teaching methods.

Students on the other hand were very positive, asserting Spaced Learning helped them learn rapidly. Perhaps the most illuminating accounts of the nature of Spaced Learning come from students, as this first hand description illustrates:

The lessons are very compressed. For example, the review of my whole Biology unit was completed in about 12 min. The nervous system, diet deficiencies, hormones and the menstrual cycle, drugs, and defence from pathogens all whiz by on slides shown at the dizzying rate of 7–8 per min. During the 10-min breaks we get physical, rather than mental, activities like basketball dribbling and teamwork games. So what happens inside your head during Spaced Learning that is different from what happens during a traditional lesson or review session? I can only answer for myself. I love rock climbing. You always have to be aware of what comes next, but you can’t consciously think about it. For me, Spaced Learning is a bit like my climbing. I don’t try to learn; I don’t write anything down, and I don’t review. It just seems as if I am seeing a movie in my mind that I have already seen before, and my understanding of the information presented becomes more precise—clearer—when I see it again. In the end, I am left with a movie in my head of the lesson, just like my memory of a climb.

My first experience of Spaced Learning came in March 2007 when my class re-took our science exams from November 2006. We only had a one hour Spaced Learning review session (which had four months of work condensed into it from the summer before). Most of us did better on the exams after an hour of Spaced Learning review, even though we did no studying at all. I went from an A, B and C to straight A’s and an A+. It was amazing.

Another student simply stated Spaced Learning “was able to hold my attention the entire time, which was rather interesting as I can sometimes be distracted and lose concentration”. These student accounts are drawn from a digital guide to Spaced Learning created for a national programme of innovative approaches in schools, Learning Futures. The publication includes video of a complete Spaced Learning session (Barratt, 2008; Bradley and Patton, 2012).

After this preliminary development stage, the research group established experiments to determine the impact of Spaced Learning as measured in the multiple choice tests for Biology in the English National Curriculum Science course. The two Biology tests (General Certificate of Secondary Education (GCSE) Biology 1 and Biology 2) covered all the curriculum test requirements for the subject. The choice of these high-stakes tests as a measure was taken as they were objective and could be linked to independent assessments of academic potential by the University of Durham CEM Center. Controlling for prior learning and validity was rigorous, and comprehensively reviewed by government bodies. The importance of the results in these high-stakes tests for students and schools was also a factor.

Students in an urban secondary school in England aged 13–15 (*n* = 440) took part in the study, and the scores of students in other schools taking the same tests were used in one condition (*n* = 1,730). Experimental groups with larger control groups were used throughout for organizational reasons and to ensure the quality of Spaced Learning was reasonably high, given the method was new to all teachers. All students taking part in the study, except those drawn from a national cohort in Condition 3, had been pre-tested for academic potential, science achievement in national and yearly tests, and in tests similar to those used in the study. Informed consent was obtained from all participants, research methods complied with national guidelines, and were approved by the Research Committee of the school’s trust.

In all Conditions control and experimental subjects were matched using data adjusted for variables including prior attainment. This was carried out using data from the University of Durham’s CEM Center created by running linear regression analyses that included comparison with standardized national data to determine individual predictions of test performance (Tymms and Coe, 2003). There were no significant differences between control and experimental groups on these measures in any condition as students taking part had potential achievement not significantly different from students nationally.

Spaced Learning was used with experimental groups in three conditions to deliver the Biology courses in the English National Curriculum. In Conditions 1 and 2 students took the second Biology course either through traditional teaching over four months (controls) or only a single Spaced Learning session of 60 min instructional time (experimentals). In condition 3 students took the first Biology course for four months with a single Spaced Learning session of 60 min replacing the end of course review session, and were tested. In all conditions the Spaced Learning session covered the whole course.

In Condition 1 students aged 13–15 were randomly assigned to experimental (*n* = 46) or control groups (*n* = 127). Condition 1 was constructed in part to restrict any learning other than through Spaced Learning, and ensuring STM was minimized or eliminated by having five days between the Spaced Learning session and the test. In order to minimize the likelihood of any prior learning on the experimental groups’ test scores, experimental groups studied the second Biology course in the academic year before the control groups, and without previously studying the first Biology course. In the following academic year, control groups studied both Biology courses for four months each, and were tested after each course.

In Condition 2 students aged 14–15 were in ability-matched groups from the beginning of the academic year, and one was randomly assigned to the experimental condition (*n* = 21) and controls were in similar-sized groups (*n* = 131). In Condition 2 students in the experimental condition had no instruction other than through Spaced Learning (as in Condition 1). In Condition 2, the normal educational context was preserved as far as reasonably possible with all students with their own group and teacher, and having completed the first Biology course before taking the second Biology course. Then, for the second Biology course, the experimental groups experienced the Spaced Learning session and were tested. Controls were taught over four months and were tested.

In Condition 3 experimental subjects aged 14–15 were taught the first Biology and Physics courses in the same teaching groups and then were tested (*n* = 115). Condition 3 was designed to test any impact of Spaced Learning after normal teaching of a course, remove the novelty of Spaced Learning without teaching, and enable more direct comparisons with another subject (Physics) and students in other schools. The normal educational context was preserved as far as possible, and the Biology course taught for four months and then the students were tested. The Physics course was taught for eight months and the students were tested as a longer period of instruction for this course was considered necessary, an approach followed in many other schools. At the end of the Physics course all students had an intensive one hour review of all course content days before the examination, as is common practice in English schools. In contrast, at the end of the Biology course, this intensive review was replaced by a single Spaced Learning session of the same duration.

Subsequent to the results of the tests for students nationally being published, it became possible to identify a cohort of similarly-aged students that had taken the same Biology and Physics tests as experimental subjects in the same month, on the same day and at the same time in the day (*n* = 1,730). This limited the impact on test scores of the circadian rhythm shift towards eveningness types and its effects on learning during adolescence (Lockley and Foster, 2012). As this shift towards eveningness increases during puberty it would have an increased impact the older the students (Roenneberg et al., 2004), and has in-day effects on cognitive function the earlier testing occurs (Carrell et al., 2011). Repeated measures data also made it possible to compare answers to questions by experimental subjects and subjects in other schools in both Biology and Physics tests. At that time, no other school in England used Spaced Learning.

## Results

Nationally standardized results of the high-stakes test for all groups in the study were analyzed by the CEM Center, comparing individuals’ predicted and achieved high-stakes test scores through linear regressions, and these data were used as the basis of results analysis.

The multi-choice test data allowed a comparison of experimental subjects’ scores and random answers. This comparison was used to assess whether any learning had occurred. Test scores for all experimental groups were significantly higher than random answers (*p* < 0.0000001, effect size = 4.97 Cohen’s *d*). This effect is duplicated in controls, leading to the unsurprising conclusion that teaching for four months had a positive impact on outcomes. In all conditions to control further for prior learning the first eight questions for the random condition were assigned correct answers (8/36 or 22%). The scale of this adjustment is indicated by comparing it with scores of students in other schools in Condition 3, where 1% fell below the standard used in the random condition by achieving less than nine correct answers.

**Figure 1:**
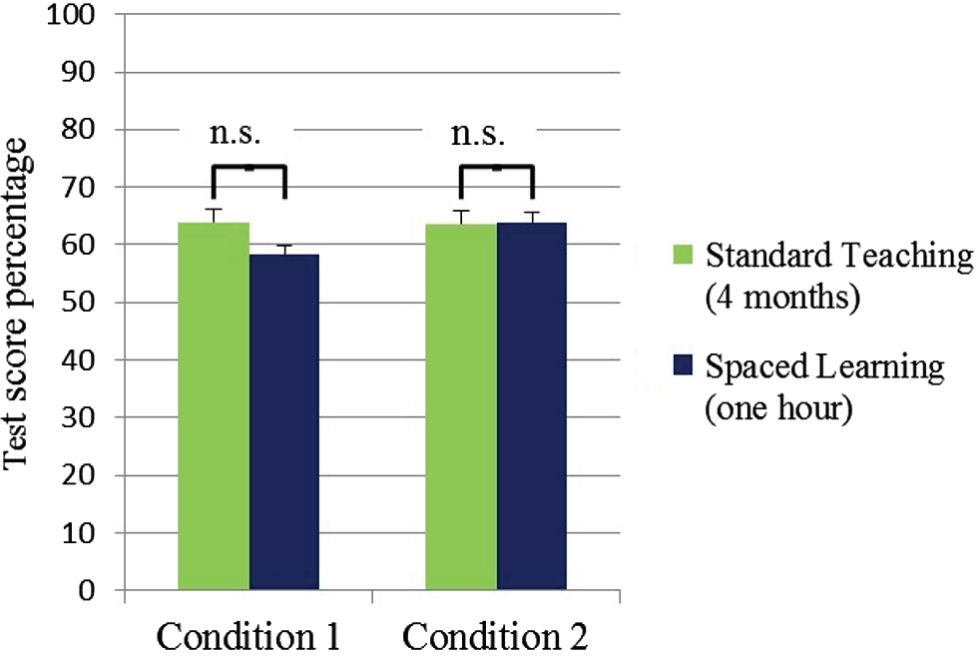
**High-stakes test scores for teaching (four months) and spaced learning (one hour).** There was no significant difference between the test scores for teaching (*N* = 127) and spaced learning (*N* = 46) groups in Condition 1. In Condition 2 there was also no significant difference between teaching test scores (*N* = 131) and spaced learning test scores (*N* = 21). All data are means ± SEM.

The test data allowed a comparison of control group scores after four months teaching and experimental group scores after an hour of Spaced Learning. In Condition 1 there were a number of restrictions intended to limit the impact of prior learning in experimental groups. These groups were in an earlier academic year, tested nine months earlier than controls, and had not studied the first Biology course. The five day gap between learning through Spaced Learning and the test in effect eliminated STM accounting for test scores. Surprisingly, the experimental groups’ high-stakes test scores after an hour’s Spaced Learning were not significantly different from controls’ test scores after four months teaching (Figure 1).

**Figure 2:**
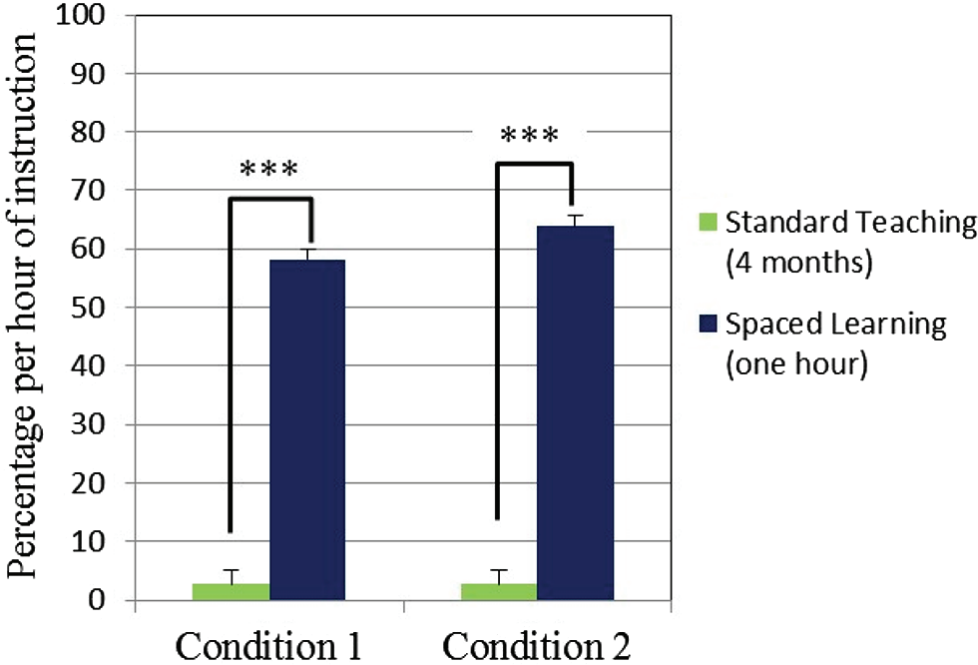
**Test score percentage increase per hour of instruction.** High-stakes test scores percentage increase per hour of instruction was significantly higher for spaced learning (*N* = 46) than for teaching (*N* = 127) in Condition 1 ***P < .00001. In Condition 2 the test score increase for spaced learning (*N* = 21) was also significantly higher than for teaching (*N* = 131) ***P < .00001. All data are means ± SEM.

In Condition 2 the context for learning was similar to the normal teaching context in academic year, prior study of the first Biology course, and teacher continuity. In this condition, experimentals’ high-stakes test scores were not significantly different from controls’ test scores (Figure 2).

In both conditions one hour of instruction through Spaced Learning had a significantly greater impact than many hours of teaching. Experimental groups’ scores were based on 60 min of instruction, and control groups’ scores on teaching over four months with 23 hours direct instruction. A measure of the impact on learning in relation to instructional time was calculated for all groups to quantify the impact difference: a test score percentage gain per hour of instruction. In both conditions, there was a highly significant difference (*p* < 0.0001) between experimental groups and control groups in learning per hour of instruction as measured by the test, confirming the intuitive judgment that one hour’s instruction replacing four months instruction demonstrates far greater efficiency in learning.

In both Conditions 1 and 2, Spaced Learning groups learned significantly faster as measured by the high-stakes test. Finally, there were no significant differences between groups of individuals within these experimental groups by sex, age, or ability after taking into account predicted and achieved high-stakes test scores.

Condition 3 test data allowed a comparison of experimental subjects’ performance in Biology and Physics, and with matched data from a national cohort. Where learning was in the normal teaching context (Physics), experimental subjects’ percent correct answers closely matched the national cohort mean (+ 0.8%, n.s.). Where learning was in the normal teaching context except for a course review in Biology being replaced by Spaced Learning of the same duration, experimental subjects’ percent correct answers were significantly higher (+ 7.6%, *p* < 0.00005, Cohen’s *d* = 0.53; Table 1). Significant differences in individual questions after Spaced Learning were found in questions in a variety of question formats and across different content areas.

**Table 1:** **Test percentage correct answers: Spaced Learning vs. course review**.

**Percentage correct answers experimental vs. national cohort**	**Mean**	**Standard Error**	**Standard Deviation**
Experimental subjects (*N* = 115)	62.84%***	1.44	15.47
National cohort subjects (*N* = 1,730)	55.24%	0.35	14.39

*Test percentage of correct answers was significantly higher when Spaced Learning replaced intensive review teaching in Biology (N = 115) compared to a national cohort (N = 1,730)*.

*** *p < 0.00005, Cohen’s d = 0.53; Two-sample t test with unequal variance. All data are means ± SEM*.

Regression analysis of experimental subject scores in Physics and Biology adjusted for ability produced a similar level of significance.

## Discussion

The results suggest there was significant impact on learning as measured in high-stakes test results using a method derived directly from studies in LTP/LTM encoding. The results also indicate long-term memories of an academic course can be created rapidly through Spaced Learning. In tests, experimental subjects exceeded random answers, matched control subject outcomes after just one hour’s Spaced Learning, and showed rapid LTP/LTM encoding at high levels of significance. If replicated consistently in further studies, this learning efficiency has significance in LTP/LTM encoding studies and significant implications for education in teaching, curriculum planning and learning resources.

Remarkably, experimental subjects acquired long-term memories of complex material as required by England’s national curriculum in one hour, apparently adjusting easily to Spaced Learning’s very intense learning and exceptional speed of delivery of the Biology courses. Students appeared to adjust to Spaced Learning effectively whether or not they were with their own teacher, group, or learning in an earlier academic year. Although there is substantial evidence that many communication systems in humans and other species operate at a very fast speed, with many elements operating in milliseconds, this does not, in itself, explain the impact of high speed Spaced Learning instruction on test scores.

The data suggest Spaced Learning is more efficient in comparison to standard teaching. There was a highly significant difference between teaching and Spaced Learning as measured by high-stakes test scores percentage increase per hour of instruction and in the duration of the instructional process taken to achieve similar test results. This has clear parallels in neuroscience studies showing very rapid memory processes in humans, and indicates both the spacing pattern used for LTP/LTM creation and the one hour duration of instruction were effective. The manipulation of time as a key variable in learning here reflects neuroscience evidence on time scales in memory processes rather than educational time scales (Tetzlaff et al., 2012). In LTP/LTM encoding studies in neuroscience, physical evidence confirming very rapid encoding of LTM occurs in time scales of minutes. That this fundamental discovery in neuroscience can be applied in education is, above all else, the central finding of this study should it be replicated in other contexts.

## Limitations and future directions

The present study was limited in scope in a number of ways. It does not directly explore the use of Spaced Learning to deliver different content in different subjects, or with students outside the 13–15 age range. The testing method was limited to high-stakes tests of a National Curriculum course in the specific context of school education. This study’s findings should be explored in different experimental designs, different contexts, with different groups, subjects of different ages, and other forms of assessment used as measures of learning. Additional preliminary data in different academic subjects and ages has been reported (Gittner, 2010). Outside formal education, a process of three repetitions spaced by 5 min spaces showed some successful outcomes in supporting learning in patients with multiple sclerosis (Goverover et al., 2011).

Research into applications Spaced Learning in different contexts may well be fruitful for particular learner groups, subjects, or ages. For example, it could be used to accelerate mastery of advanced topics with able students, or rapidly create LTM for students who are easily distracted. It could also be deployed in many different ways, including sequences of Spaced Learning instruction to assist with rapid acquisition of knowledge, skills, or information. It could also be used in combination with other less common forms of learning, such as Enquiry-based Learning, as in the Learning Futures programme.

There are three areas of research into LTM processes that appear to have implications in education arising from the importance of time in LTM encoding, consolidation and retrieval. The first area is explored in this study: the timing protocols that are effective in triggering LTP and LTM encoding. Encoding long-term memories requires more research into the range of times that can be used for spacing, the number of instructional repetitions, and identifying other protocols that induce LTP and LTM encoding. For example, LTP can be triggered in rats in very short spaces by alternating hidden and visual cues (Feldman et al., 2010), using 10 min spaces without stimuli (Morris, 2003; Fields, 2005), and spaces of an hour or more (Kramár et al., 2012). Evidence from other species (Jin et al., 2011) and recent LTP research cast further light on these issues (Redondo and Morris, 2011). It also appears that other LTM processes may operate in different time scales: for encoding (minutes or hours), consolidation (days) and retrieval and maintenance (months or years). The clarification of these issues, and methods developed from the research, could be of great benefit in education.

The second area for further research is adjusting educational practice to protect LTM consolidation processes, particularly in sleep. Sleep enhances LTP and LTM (Stickgold, 2005; Diekelmann and Born, 2010), and sleep deprivation can disrupt learning in adolescence (Giedd, 2009; Holm et al., 2009; Wang et al., 2011). Sleep functions are active processes that can consolidate, relocate and integrate memories (Lockley and Foster, 2012). Within sleep cycles, both Slow Wave Sleep (Chauvette et al., 2012) and Rapid Eye Movement (REM) sleep (Grosmark et al., 2012) appear to have different memory functions, and this may be the basis of a more specific theory of sleep’s function in memory processes (Wagner et al., 2004; Mednick et al., 2011). Interestingly in an educational context, it appears cueing the importance of a memory for future use in a test appears to increase the likelihood of that memory being consolidated (Wilhelm et al., 2011). The use of devices before sleep that emit high levels of blue light disrupt sleep, and this should inform the timing of online learning in education and the use of electronic devices in the last hour before sleep. The most important issue is the timing of education institutions’ days, as these are known to conflict with the time shift towards eveningness during adolescent circadian rhythm changes. The mismatch between educational timings and circadian neuroscience evidence strongly suggest major readjustment of current educational practice is required (Czeisler, 2009; Hagenauer et al., 2009; Lockley and Foster, 2012). There remains an urgent need to determine the times for education institutions that are appropriate at different ages in adolescence, given the slow speed of change in sleep patterns during puberty (Roenneberg et al., 2004). Synchronizing educational times to adolescent circadian rhythms within the day may enhance other memory processes, as studies suggest both LTM encoding and retrieval in tests for adolescents are better at later times in the day (Hahn et al., 2007; Carrell et al., 2011).

The third area for further research is determining the timing protocols that are effective in enhancing retrieval and maintenance of long term memory. Retrieval of LTM is a distinct memory process (Gabrieli et al., 1997) where spacing retrieval practice is known to be effective in improving retrieval, and the greater impact of retrieval practice over further learning practice appears to be a key factor in improving retrieval (Karpicke and Roediger, 2008). The timing of formal tests (Carpenter et al., 2008, 2012) and studies aiming to identify optimal patterns of retrieval practice (Karpicke and Blunt, 2011) are fruitful approaches. These seem areas for further research where neuroscience may be able to contribute to our understanding of retrieval memory processes identified in psychology. The use of tests, their function in improving retrieval, and the optimal pattern of spaces for LTM retrieval practice are arguably the most important issue for education. Karpicke and his colleagues (Karpicke and Roediger, 2008; Karpicke and Bauernschmidt, 2011; Karpicke and Blunt, 2011) have made great progress in this area in the last five years, and there is a growing body of research into retrieval processes in the neuroscience literature. Recently researchers developed a spaced retrieval method with three repeated tests that produced a 200% improvement in long-term retention relative to repeated retrieval trials with no spacing between tests (Karpicke and Bauernschmidt, 2011). Further research in this area, and trials in education, could prove extremely valuable.

Research in neuroscience on learning and memory should inform in education policy and practice, though this process can be complex (Goswami, 2006; Meltzoff et al., 2009; Royal Society, 2011). Spaced Learning may be another demonstration of research in neuroscience leading to improved educational practice (Gabrieli, 2009), and making a contribution to improving educational understanding of the creation long-term memories.

## Conflict of interest statement

The authors declare that the research was conducted in the absence of any commercial or financial relationships that could be construed as a potential conflict of interest.
